# Multiple Tophaceous Gout of Hand with Extensor Tendon Rupture

**DOI:** 10.1155/2017/7201312

**Published:** 2017-12-20

**Authors:** Haruki Tobimatsu, Masanori Nakayama, Yu Sakuma, Hitoshi Imamura, Koichiro Yano, Hiroko Itagaki, Katsunori Ikari

**Affiliations:** ^1^Department of Orthopedic Surgery, Institute of Rheumatology, Tokyo Women's Medical University, 10-22 Kawada-cho, Shinjuku-ku, Tokyo 162-0054, Japan; ^2^Department of Surgical Pathology, Tokyo Women's Medical University, 8-1 Kawadacho, Shinjuku-ku, Tokyo 162-8666, Japan

## Abstract

A 45-year-old man presented with painless subcutaneous masses bilaterally on his hands and loss of motion or contracture of the fingers. Initially, drug therapy to reduce the serum uric acid was administered and was expected to reduce the tophi. However, during observation at the clinic, spontaneous rupture of an extensor tendon occurred, and surgical repair of the tendon and resection of the masses were performed. Surgical exploration of the right hand showed hypertrophic white-colored crystal deposits that both surrounded and invaded the extensor digitorum communis of the index finger, which was ruptured. Histopathologic examination of the specimen demonstrated findings consistent with gouty tophi. Tophaceous gout can induce a rupture of tendons during clinical observation, and surgical resection of the tophi might be needed to prevent ruptures.

## 1. Introduction

Gout is characterized by hyperuricemia and attacks of acute synovial inflammation secondary to the deposition of sodium urate crystals [1–3]. With recent improvements in hyperuricemia treatment, gouty tophi are found rarely clinically. However, poor control of hyperuricemia can result in a heavy tophus in some cases [1–3]. In general, spontaneous tendon rupture in the hand occurs in association with rheumatoid arthritis, distal radius fractures, or osteoarthritis of the distal radioulnar joint [4]. There have been only a few reports of tendon rupture in the hand or wrist due to gout. We report here an unusual case involving a spontaneous extensor tendon rupture secondary to extensive tophaceous gout affecting the hand.

## 2. Case Presentation

The patient was a 45-year-old man with a chief complaint of multiple masses on his fingers and hands. The first mass was noticed at 26 years of age, and although the patient was diagnosed with gout by his doctor, it was not treated. His first examination in our hospital was at 31 years of age, and drug treatment for hyperuricemia was administered. However, about five years later, he stopped this treatment by himself, and there are no records of consultations since then. At 45 years of age, the patient was reexamined at our hospital wishing to undergo surgery for tophi on both hands.

**Figure 1:**
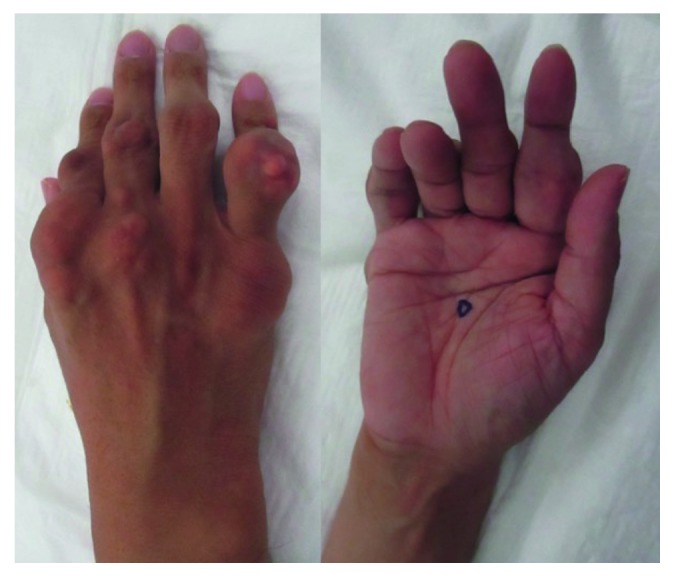
A photograph of the patient's right hand. There were several subcutaneous nodules in the hand and digits. The index finger was not able to extend actively at the metacarpophalangeal joints.

**Figure 2:**
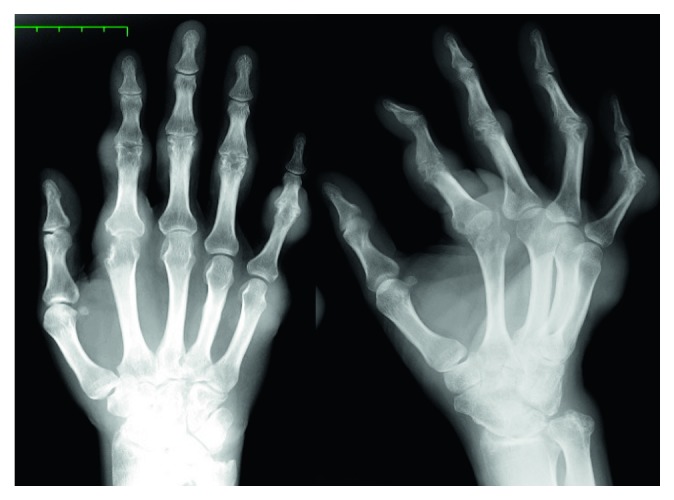
Preoperative radiograph of the right hand showing gouty arthritis and inflammatory osteolysis with numerous calcified soft tissue swellings, especially in the proximal interphalangeal joints of the index, middle, and little fingers.

Physical examination revealed multiple tophi on both hands (Figure 1) and similar tophi bilaterally on the elbows, knees, toes, and ankle joints. At that time, the PIP joint of the right index finger was contracted. MP joint dislocation was suspected on the palmar side, but the joint could be extended fully. Blood tests at the time of reexamination showed the uric acid (UA) level was elevated to 11.2 mg/dl. Plain radiography showed multiple soft tissue shadows, indicative of tumors and bone erosion in the PIP joints of the four fingers, and the MP joint of the index finger (Figure 2). Palmar dislocation of the MP joint of the index finger was observed as well. Based on the clinical findings, the patient was diagnosed with gouty tophi and oral administration of Febuxostat (40 mg per day) for hyperuricemia was started to reduce the level of serum uric acid. It was expected that decreasing the uric acid would reduce the tophi.

**Figure 3:**
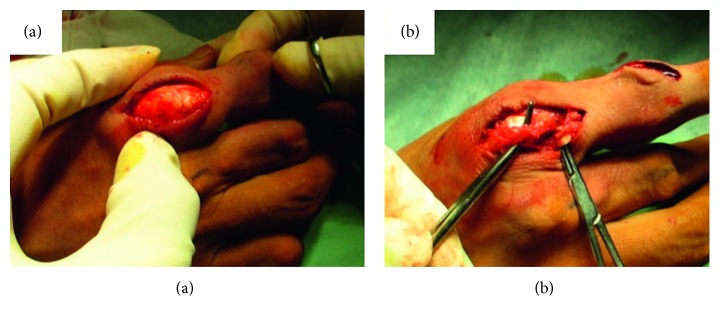
(a) Intraoperative findings. Hypertrophic white crystal deposits consistent with gouty tophi are seen surrounding the extensor digitorum communis of the index finger and extensor indicis proprius tendon. (b) After debridement of the tophi, the extensor indicis proprius was found to be intact but slightly elongated.

**Figure 4:**
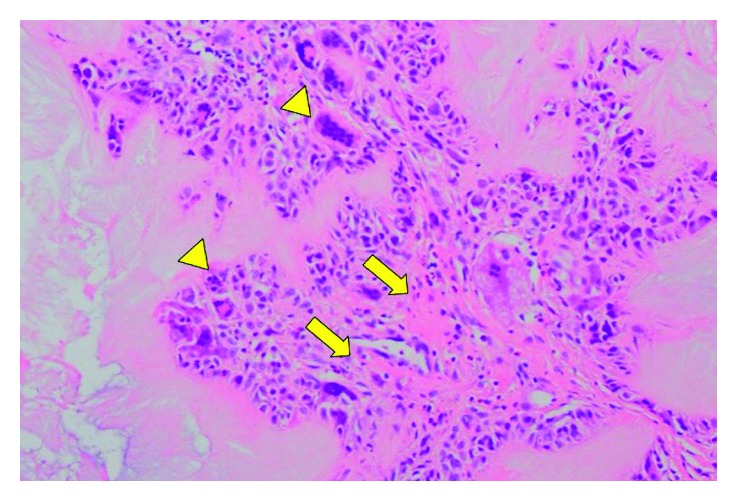
Photomicrograph of a section from the resected tissue specimen showing an eosinophilic amorphous tophaceous deposit surrounded by chronic inflammatory cells and multinucleated giant cells (arrow heads) and surrounded by fibrillations around them (arrows) (H&E stain, ×100).

**Figure 5:**
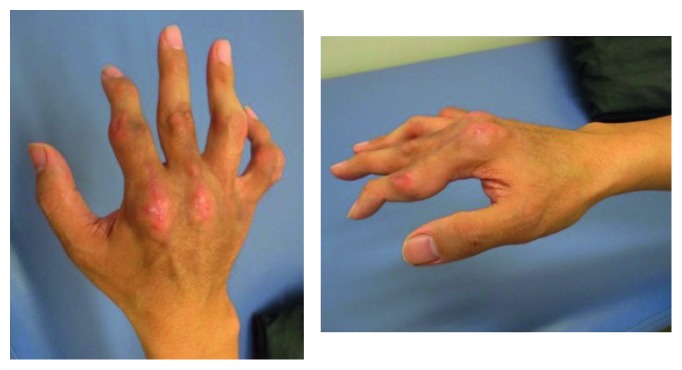
Photograph of the right hand 6 weeks following surgery showing significant reduction of the tophi.

After about two months, the patient complained about his index finger disorder and was referred to our clinic. His right index finger was difficult to extend actively, but it could be extended passively. An extensor tendon rupture of the index finger was suspected, and surgery for resection of the mass and repair of the extensor tendon was planned. Perioperative findings showed that the extensor digitorum communis of the index finger in the MP joint was invaded by a mass, and its margins were identified proximally and distally. The extensor indicis muscle tendon was found on the ulnar side of the mass and was not ruptured (Figure 3). Following resection of the mass, the extensor indicis muscle tendon was returned to its original position, and the patient was able to extend his index finger. Therefore, it was not necessary to repair the extensor digitorum communis. We also resected the masses in the PIP joints of the index, middle, and little fingers, as well as in the MP joints of the middle and little fingers. Macroscopic findings showed the masses to be like gouty tophi. Pathologically, crystalline masses surrounded by palisades of multinucleate giant cells and lymphocytes, together with fibrosis, also were found (Figure 4). The hand was immobilized with a splint for two weeks, followed by motion training. In addition, the patient continued to be administered Febuxostat (40 mg per day) for hyperuricemia, and the UA level six months after surgery was 5.5 mg/dl. Six months after surgery, there was no recurrence of the tophi, and while there was some limitation in the extension of the MP joint of the index finger, which had an arc of motion of 60° (range −20° to 80°), the patient was satisfied with the cosmetic and finger motion improvements (Figure 5).

## 3. Discussion

Gout is an inflammatory disease characterized by an elevated serum urate concentration and recurrent flares, including painful, hot, red, and swollen joints and surrounding tissue [5]. In general, hyperuricemia is defined as serum UA exceeding 7.0 mg/dl. Urate deposits eventually produce chalky masses called gouty tophi in patients with a long history of the disease [5]. Formerly, about 70% of gouty tophi cases occurred in patients with serum UA exceeding 11.0 mg/dl [6]. However, the occurrence of gouty tophi decreased due to the recent development of antihyperuricemic drugs. According to a survey in USA in 2010, gouty tophi occurred in 12% of patients who had hyperuricemia [7]. In France, between 2008 and 2009, gouty tophi occurred in 19.4% of those with hyperuricemia [8]. In Japan, the occurrence rate was smaller than that in Western countries; gouty tophi occurred in 5% of 422 hyperuricemic cases referred to our institute between 2007 and 2008 [9].

Gouty tophi are usually treated conservatively using drugs [10]. Surgical treatment is performed rarely because tophi are expected to diminish with drug therapy, and there are complications of surgery, such as infection, gout attacks, or excretion of uric acid crystals [10]. The traditional indications for surgery for gouty tophi are described as (1) functional, which includes excision to allow wearing of clothing and gloves, restoration of motion, and joint stability; (2) symptomatic, which includes reduction of pain, decompression of nerves, control of infection, discharging sinus, and skin ulceration; and (3) cosmetic restoration, especially for tophaceous gout in the hand and wrist [11]. In our case, surgery for tophi was performed for functional and cosmetic reasons.

There have been reports that gouty tophi in the upper extremity were surgically resected because of entrapment neuropathy [12], flexion deformity of the finger [13], or tendon ruptures. To the best of our knowledge, there have been only 5 reports of hand or wrist tendon ruptures due to gout [4, 14–17]. A few extensor tendon rupture cases have been reported [4, 14, 15]. In one case, a painless subcutaneous tumor caused rupture of the common digital extensor muscle tendon followed by poor extension of the ring finger [4]. There are also few flexor tendon rupture cases [16, 17]. One report showed a superficial digital flexor tendon that was ruptured as a result of widespread gouty tophi in the carpal tunnel [16]. During the observation of gouty tophi in a hand, it is very important to remember that it can affect tendons, and they may be ruptured.

The decision regarding whether to remove crystals from inside the tendons or ligament attachment sites is difficult because it is often necessary to destroy those tissues to remove crystals completely. Therefore, crystals cannot be removed frequently [1, 2]. Previous reports show that UA crystals are often excreted postoperatively [1, 2]. This suggests that, depending on the degree of tendon parenchymal degeneration, control of hyperuricemia may prevent tendon ruptures.

## 4. Conclusion

We performed surgical treatment for a case of multiple gouty tophi in the fingers complicated by rupture of the extensor tendon of the index finger. It should be recognized that gouty tophi might cause tendon rupture, and control of serum UA is important to prevent tendon rupture.
